# Flavonoids increase melanin production and reduce proliferation, migration and invasion of melanoma cells by blocking endolysosomal/melanosomal TPC2

**DOI:** 10.1038/s41598-021-88196-6

**Published:** 2021-04-19

**Authors:** Ponsawan Netcharoensirisuk, Carla Abrahamian, Rachel Tang, Cheng-Chang Chen, Anna Scotto Rosato, Wyatt Beyers, Yu-Kai Chao, Antonio Filippini, Santiago Di Pietro, Karin Bartel, Martin Biel, Angelika M. Vollmar, Kaoru Umehara, Wanchai De-Eknamkul, Christian Grimm

**Affiliations:** 1 Walther Straub Institute of Pharmacology and Toxicology, Faculty of Medicine, Ludwig-Maximilians-University, Munich, Germany; 2 Department of Biochemistry and Microbiology/Pharmacognosy, Faculty of Pharmaceutical Sciences, Chulalongkorn University, Bangkok, Thailand; 3 Department of Pharmacy, Center for Drug Research, Ludwig-Maximilians-University, Munich, Germany; 4 Department of Biochemistry and Molecular Biology, Colorado State University, Fort Collins, CO USA; 5 Department of Anatomy, Histology, Forensic Medicine and Orthopedics, Unit of Histology and Medical Embryology, Sapienza University of Rome, 00161 Rome, Italy; 6 Yokohama University of Pharmacy, Yokohama, Japan

**Keywords:** Cancer, Chemical biology, Ecology

## Abstract

Two-pore channel 2 (TPC2) resides in endolysosomal membranes but also in lysosome-related organelles such as the melanin producing melanosomes. Gain-of-function polymorphisms in hTPC2 are associated with decreased melanin production and blond hair color. Vice versa genetic ablation of TPC2 increases melanin production. We show here an inverse correlation between melanin production and melanoma proliferation, migration, and invasion due to the dual activity of TPC2 in endolysosomes and melanosomes. Our results are supported by both genetic ablation and pharmacological inhibition of TPC2. Mechanistically, our data show that loss/block of TPC2 results in reduced protein levels of MITF, a major regulator of melanoma progression, but an increased activity of the melanin-generating enzyme tyrosinase. TPC2 inhibition thus provides a twofold benefit in melanoma prevention and treatment by increasing, through interference with tyrosinase activity, the synthesis of UV blocking melanin in melanosomes and by decreasing MITF-driven melanoma progression by increased GSK3β-mediated MITF degradation.

## Introduction

Wile many cancer incidences are falling, the incidence rate of malignant melanoma is rising at a rate of 3–7% in most European countries versus 2.6% in the US, and is expected to further rise. There are about 100.000 new cases per year in Europe and the US, each, with approximately 22.000 deaths per year in Europe and 7.000 in the US^1,2^. Statistics in the US indicate that melanoma is more than 20 times more common in whites than in African Americans. When discovered early, melanoma can be surgically removed and patients have a high chance of being cured. However, when metastases have already formed, the prognosis is generally very poor, going along with a strongly decreased life expectancy. Patients then survive only 6–9 months on average after diagnosis, highlighting the importance of early diagnosis but also the need for new effective melanoma treatments. Melanocytes produce melanin in their melanosomes and most melanoma cells also still make melanin. Hence, most melanoma tumors appear black or brown while some do not make melanin anymore and can then appear pink, tan, or even white. Individuals with a higher ratio of yellow pheomelanin to brown eumelanin in their skin and hair, i.e. blondes and redheads have a greater risk for melanoma than black or brown haired individuals (by a factor of 2–4). The pheomelanin/eumelanin ratio accounts for some of this risk^3^.

Ambrosio et al. (2016) have recently shown that knockout of two-pore channel TPC2 in human MNT-1 melanoma cells elicits a strong increase in pigment content and that this effect can be reversed by transient overexpression of TPC2-GFP^4^. Vice versa, Sulem et al. (2008) have shown that certain TPC2 polymorphisms, rs35264875 (encoding TPC2M484L) and rs3829241 (encoding TPC2G734E) are associated with reduced pigmentation and a higher probability for blond hair in humans^5^. Chao et al. (2017) investigated the functional effects of these variations on the channel properties using endolysosomal patch-clamp electrophysiology and found that both polymorphisms are gain-of-function (GOF) variants^6^. Furthermore, it has been shown recently that pharmacological or siRNA mediated inhibition of TPC2 abrogates migration of cancer cells and the formation of metastases^7^. However, a more detailed mechanistic understanding of how TPC2 activity and expression in melanosomes on the one hand and endolysosomes on the other hand affect melanoma cells is lacking. Here, we used MNT-1 human melanoma cells to assess the effect of genetic ablation or pharmacological inhibition of TPC2 on proliferation, migration, and invasion as well as melanin production. Surprisingly, we found that melanoma cell proliferation, migration, and invasion are inversely correlated with TPC2-dependent melanin production as loss of TPC2 increases melanin content but decreases proliferation/migration/invasion. This is possible due to independent mechanisms: via regulation of MITF (microphthalmia-associated transcription factor) protein levels through interference with endolysosomal activity of TPC2 and endolysosomal GSK3β degradation on the one hand and on the other hand via MITF-independent regulation of tyrosinase activity by TPC2 in melanosomes. Similar effects were found with novel, flavonoid based inhibitors of TPC2, corroborating a new treatment option for melanoma using TPC2 as a pharmacological target. Flavonoids, previously proposed anti-cancer agents thus emerge as direct inhibitors of TPC2 and the higher risk for blond haired individuals or individuals with light pigmentation to develop melanoma may be directly correlated to TPC2 activity and GOF variation.

## Results

### Human TPC2^−/−^ melanoma cells show reduced proliferation, migration, and invasion but increased melanin production and tyrosinase activity

**Figure 1:**
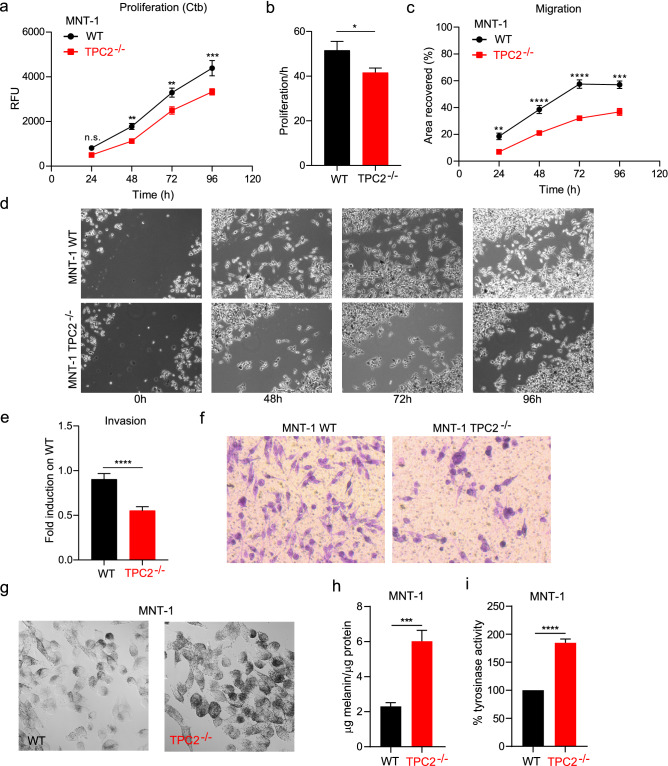
Proliferation, migration, and invasion in WT and TPC2^**−/−**^ MNT-1 cells. Proliferation was assessed using the CellTiter-Blue (Ctb) assay for the different time-points subtracted from the 0 h value reading of cells. Viable, metabolically active cells undergo a reduction reaction of resazurin to resorufin, while non-viable cells do not. Migration was assessed using the wound healing scratch assay, and invasion using transwell chambers in MNT-1 melanoma cells. (**a**) Genetic ablation of TPC2 in MNT-1 cells shows significant reduction of proliferation over 96 h. (**b**) Rate of proliferation per hour shows significant reduction in TPC2^**−/−**^ cells. (**c**) Migration in TPC2^**−/−**^ cells is significantly slower compared to WT MNT-1 cells, monitored for 24, 48, 72, and 96 h. (**d**) Representative images of wound healing monitored in WT and TPC2^**−/−**^ cells at different time-points. (**e**) and (**f**), MNT-1 TPC2^**−/−**^ are significantly less invasive than WT cells after 24 h. Statistical analysis (**e**) and representative Figures of WT and TPC2^**−/−**^ MNT-1 cells are shown (**f**). Cells were allowed to migrate into transwell chambers for 24 h. (**g**) Laser scanning microscope images visualizing the differences between WT and TPC2^**−/−**^ MNT1 cells in melanin content. (**h**) Quantification of melanin content in WT and TPC2^**−/−**^ MNT1 cells normalized to total protein content. (**i**) Tyrosinase activity was determined by the rate of dopachrome formation. The activity was expressed as percentage of WT cells. Statistical significance was determined by either two-way ANOVA followed by Bonferroni multiple comparisons test (**a**, **c**) or Student’s t-test (**b**, **e**, **h**, **i**). Shown are mean values ± SEM, (n = 3, each). *P < 0.05, **P < 0.01, ***P < 0.001, ****P < 0.0001.

Genetic ablation or inhibition of TPC2 has been demonstrated before to affect cancer cell migration and the formation of metastases^7,8^ as well as neoangiogenesis^9^. We show here that knockout of TPC2 in MNT-1 human melanoma cells results in significant decrease in melanoma cell proliferation, migration, and invasion (Fig. 1a–f). Ambrosio et al. (2016) have recently reported that TPC2 knockout elicits a strong increase in pigment content in MNT-1 cells^4^. Consistently, siRNA-mediated knockdown of TPC2 was also found to cause a substantial increase in melanin content in MNT-1 cells and primary human melanocytes^4^. We recapitulated these findings and confirmed the strong increase in melanin production in TPC2^**−/−**^ cells (Fig. 1g,h). We further found a significant increase in the activity of tyrosinase, which is the key enzyme responsible for efficient melanin production with a pH optimum at 6.8 (Fig. 1i). At the same time, we also found increased protein levels of tyrosinase in TPC2^**−/−**^ MNT-1 cells while transcript levels were unchanged (Fig. S1a-d and S1g). In contrast to tyrosinase, expression levels of other proteins involved in melanogenesis or regulated by MITF such as Dct (dopachrome tautomerase, TYRP2), Rab27a, or PMEL (premelanosome potein) were unchanged (Fig. S1c-f).

### Flavonoids from a Southeast Asian plant extract affect melanin production in melanoma cells

**Figure 2:**
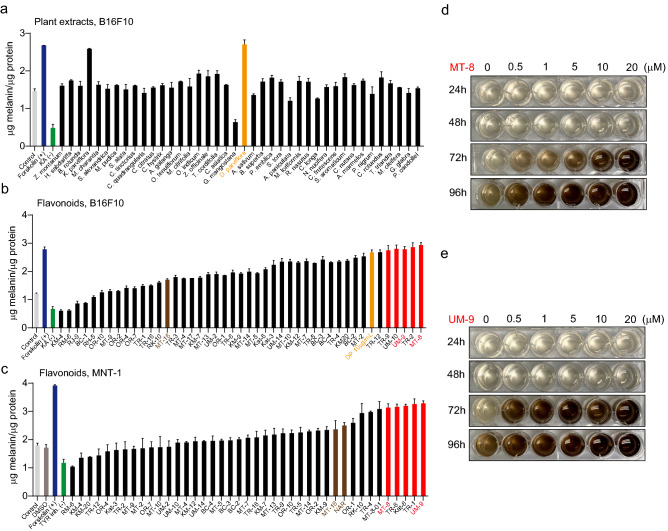
Flavonoid compound screening in B16F10 and MNT-1 cells using effect on melanin content as read-out. (**a**) Effect of different plant extracts including D. parviflora extract on the melanin content of B16F10 cells. Forskolin was used as positive control. KA (Kojic acid = 5-Hydroxy-2-(hydroxymethyl)-4H-pyran-4-one) was used as negative control. Data are shown as mean values ± SD (n = 3, each). (**b**) Effect of different flavonoids on the melanin content of B16F10. Forskolin was used as positive control. KA was used as negative control. MT-16 = NAR = naringenin was shown before to block TPCs. The data are shown in the sequence of ascending effect. Top five hits are highlighted in red and *D. parviflora* (DP) extract is highlighted in yellow. Data are shown as mean values ± SD (n = 3, each). (**c**) Effect of different flavonoids on the melanin content of MNT-1 cells. Forskolin was used as positive control. 4-Butyl-resorcinol (TYR inh.) was used as negative control. The data are shown in the sequence of ascending effect. Top five hits are highlighted in red. In both B16F10 and MNT-1 cells the compounds UM-9 and MT-8 were consistently found to be among the top 5 hits. MT-16 and commercially available NAR (cat. #67604-48-2; Sigma) are highlighted in brown. Data are shown as mean values ± SD (n = 3, each). In all experiments the following concentrations of the compounds were used: Forskolin (10 µM), TYR inh. (10 µM), KA (10 µM), flavonoids (20 µM). In a-c mean values ± SEM are shown, (n = 3, each). (**d**) and (**e**) Examples of experiments showing the time and concentration dependent effects of MT-8 (**d**) and UM-9 (**e**) on melanin content in B16F10 cells. For quantification see Fig. S2c-f.

In an unbiased approach to identify compounds that affect melanin production in melanoma cells, we screened several plant extracts in B16F10 mouse melanoma cells. An extract prepared from *Dalbergia parviflora* (*D. parviflora*), also called Akar Laka which is mainly found in lowland tropical areas, in particular in Myanmar, Thailand, Malaysia, Indonesia, and the Philippines showed the strongest effect alongside *Kaempferia parviflora* (*K. parviflora*), also called Thai ginseng or Thai black ginger, a native plant of Thailand (Fig. 2a). Subsequently, 44 different flavonoid compounds were isolated from *D. parviflora* (Fig. S2) and tested for their effects on melanin production in B16F10 mouse melanoma cells (Fig. 2b; Fig. S3a and 3b). Among the top five hits with the strongest effect on melanin generation were MT-8, an O-methylated isoflavone, also called pratensein, and UM-9, a tri-O-methylated isoflavan, also called duartin. Based on the results obtained in the melanin content assay, the following chemical features were identified to be optimal for the isoflavone group (e.g. MT-8): Aryl ring A should be dihydroxy-substituted at positions 5 (-OH) and 7 (-OH), and the aromatic ring B should be methoxy-substituted at position 4′. For the isoflavan group (e.g. UM-9), ring A should be one hydroxy-substituted at position 7 and one methoxy-substituted at position 8, with ring B being dimethoxy-substituted at positions 4′ and 2′ (or 3′). Subsequently, the screening was replicated in human melanoma cells (MNT-1), where UM-9 and MT-8 were again found to be among the top five hits (Fig. 2c). The effects of UM-9 and MT-8 were found to be concentration and time dependent. The most prominent effects were seen at day 3–4 when using a concentration of 10–20 µM in B16F10 cells (Fig. 2d,e; Fig. S3c-f). Similar optimal concentrations were found for MNT-1 cells (Fig. S3g-h).

### Flavonoids block TPC2 activity in endolysosomal patch-clamp experiments

**Figure 3:**
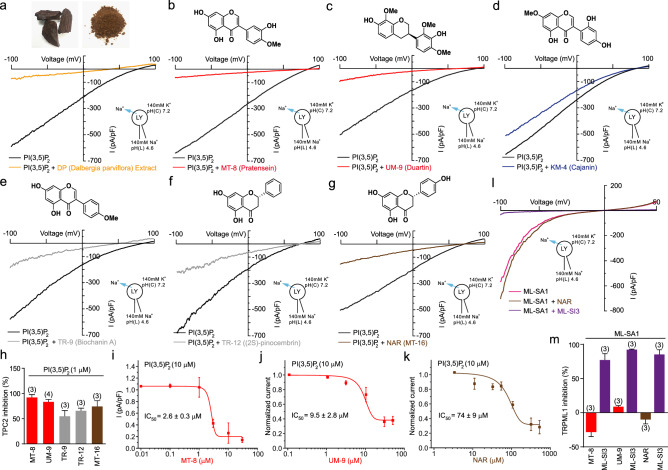
Flavonoids as inhibitors of TPC2 channel activity. (**a**) Heartwood and extract from *D. parviflora* (upper panel), and endolysosomal patch-clamp measurement demonstrating effect of the extract (10 µg/ml) to block TPC2 stimulated by PI(3,5)P_2_ (1 µM). Shown is a representative PI(3,5)P_2_-evoked current from enlarged endolysosomes isolated from HEK293 cells stably expressing human TPC2 (hTPC2). Recordings were carried out using standard bath and pipette solutions and applying ramp protocols (− 100 mV to + 100 mV over 500 ms) every 5 s at a holding potential of -60 mV. (**b**–**g**) Similar recordings as shown in a using different flavonoids (10 µM, each) to block TPC2. Structures of the respective test compounds are shown on top of the I–V traces. (**h**) Shown are average current densities (mean ± SEM) at − 100 mV of experiments as shown in **b**–**g**. (**i**–**k**) Effect-response relationships of MT-8, UM-9, and NAR using 10 µM PI(3,5)P_2_ for activation. (**l**–**m**) Data showing no blocking effect of MT-8 or NAR on TRPML1 (activation with 10 µM ML-SA1). As a positive control TRPML blocker ML-SI3 was used.

The flavonoid naringenin (NAR) has been shown before to block TPC2^10^. We therefore tested NAR and other candidate blockers including MT-8 and UM-9 in endolysosomal patch clamp experiments to assess their activity on hTPC2 inhibition following activation with the endogenous endolysosomal membrane phosphoinositide PI(3,5)P_2_. In these experiments, using hTPC2 stably overexpressed in HEK293 cells, *D. parviflora* extract, MT-8 and UM-9 were found to exhibit strong inhibitory effects on hTPC2 (Fig. 3a–c,h). In contrast, KM-4 which showed very weak effects on pigmentation, showed also very weak inhibitor activity on hTPC2 (Fig. 3d). TR-9 and TR-12 which showed variable effects on melanin generation also proved to be less efficacious inhibitors of hTPC2 (Fig. 3e–f,h). NAR (MT-16), which had a comparably strong effect in human MNT-1 cells (weaker in mouse B16F10 cells) on pigmentation, was confirmed as an inhibitor of hTPC2 (Fig. 3g–h). IC_50_ values indicate however that NAR is less potent than UM-9 and MT-8 (IC_50_ (NAR) = 74 ± 9 µM; IC_50_ (MT-8) = 2.6 ± 0.3 µM; IC_50_ (UM-9) = 9.5 ± 2.8 µM) (Fig. 3i–k). NAR was also less potent in increasing the relative melanin content (Fig. S3i). In contrast to TPC2, the endolysosomal cation channel hTRPML1, stably overexpressed in HEK293 cells, was not blocked by flavonoids but instead by the previously reported TRPML blocker ML-SI3^11^ (Fig. 3l–m).

### TPC2-inhibiting flavonoids increase melanin production and tyrosinase activity in human melanoma cells in a TPC2-dependent manner

**Figure 4:**
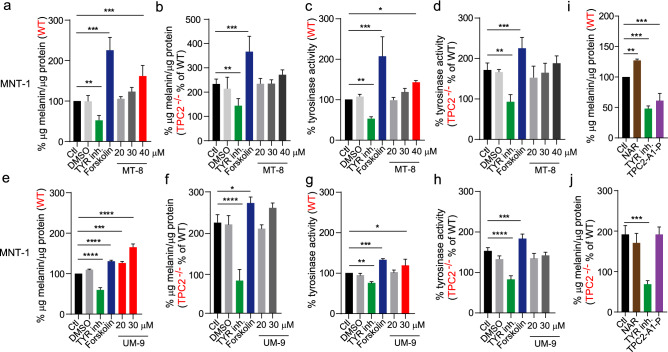
Melanin production and tyrosinase activity in flavonoid treated WT and TPC2^**−/−**^ MNT-1 cells. (**a**) and (**b**) Melanin content in WT and TPC2^**−/−**^ MNT1 cells after treatment with different concentrations of MT-8 (20, 30 and 40 µM) or DMSO, and with tyrosinase inhibitor (10 µM) and forskolin (10 µM) as negative and positive controls, respectively. (**c**) and (**d**) Tyrosinase activity in WT versus TPC2^**−/−**^ MNT1 cells after treatment with MT-8 in different concentrations. (**e**) and (**f**) Melanin content in WT and TPC2^**−/−**^ MNT1 cells after treatment with different concentrations of UM-9 (20 and 30 µM) or DMSO, and with tyrosinase inhibitor (10 µM) or forskolin (10 µM) as negative and positive controls. (**g**) and (**h**) Tyrosinase activity in WT versus TPC2^**−/−**^ MNT1 cells after treatment with UM-9 in different concentrations. (**i**) and (**j**) Melanin content in WT and TPC2^**−/−**^ MNT-1 cells after treatment with naringenin (NAR; 50 µM), tyrosinase inhibitor (10 µM), or TPC2-A1-P (TPC2 activator; 50 µM). Statistical significance was determined by one-way ANOVA followed by Dunnett ‘s multiple comparisons test. Shown are mean values ± SEM, (n = 3, each). *P < 0.05, **P < 0.01, ***P < 0.001, ****P < 0.0001.

Next, we further assessed the effect of the TPC2 inhibitors MT-8 and UM-9 on melanin generation and tyrosinase activity in MNT-1 WT versus TPC2^**−/−**^ cells. Forskolin^12^ and the tyrosinase inhibitor 4-Butyl-resorcinol (TYR inh.)^13^ were used as positive and negative controls, respectively. TPC2^**−/−**^ cells were used to assess specificity of the compound effects. MT-8 significantly increased melanin production and tyrosinase activity in WT MNT-1 cells while no significant increase was found in TPC2^**−/−**^ MNT-1 cells, suggesting effects were TPC2-dependent (Fig. 4a–d). UM-9 also significantly increased melanin production and tyrosinase activity in WT MNT-1 cells while no significant increase was found in TPC2^**−/−**^ MNT-1 cells, suggesting effects were again TPC2-dependent (Fig. 4e–h). Vice versa, activation with the novel lipophilic small molecule agonist TPC2-A1-P^14^ resulted in the opposite effect, reducing melanin production similar to TYR inhibitor with no effect in TPC2^**−/−**^ MNT-1 cells (Fig. 4i,j). NAR was also confirmed, like MT-8 and UM-9 to increase melanin production in a TPC2 dependent manner (Fig. 4i,j).

### TPC2-inhibiting flavonoids reduce melanoma cell proliferation, migration, and invasion in a TPC2-dependent manner

**Figure 5:**
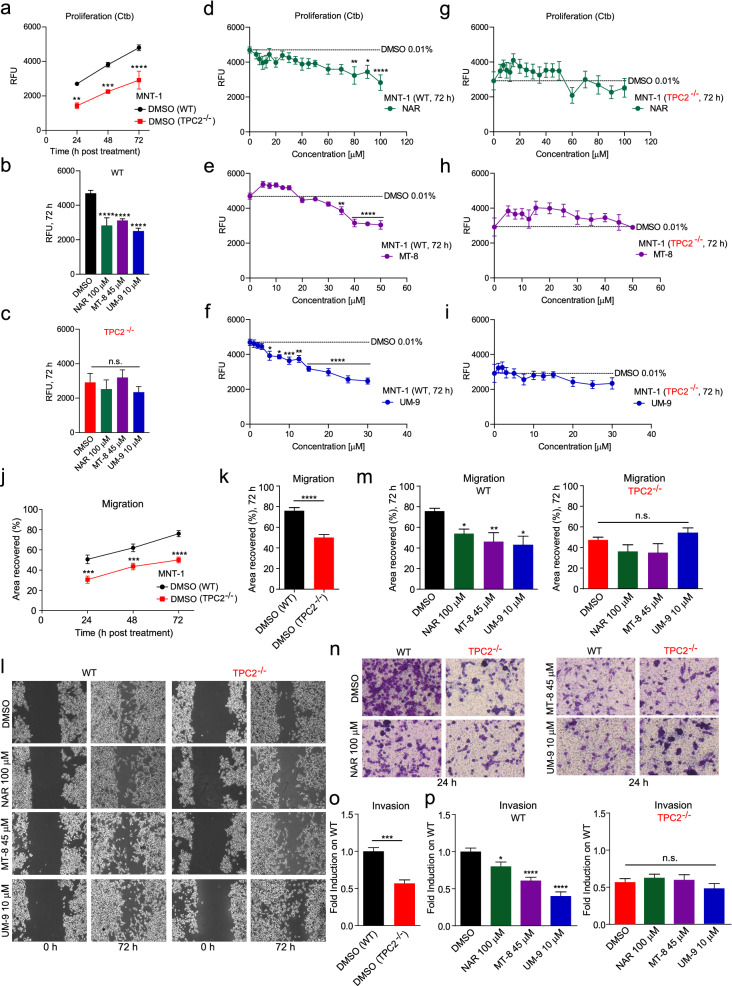
Proliferation, migration, and invasion in flavonoid treated WT and TPC2^**−/−**^ MNT-1 cells. (**a**) Proliferation of WT and TPC2^**−/−**^ MNT-1 cells after treatment with DMSO control for 24, 48, and 72 h. (**b**) Anti-proliferative effect of UM-9 (10 µM), MT-8 (45 µM), and NAR (100 µM) on MNT-1 WT cells compared to DMSO control 72 h post treatment. (**c**) Application of the flavonoids as above to TPC2^**−/−**^ MNT-1 cells shows no significant difference as compared to vehicle control on proliferation at 72 h post treatment. (**d**–**i**) Effect of NAR, MT-8, or UM-9 treatment for 72 h in MNT-1 WT (d-f) and TPC2^**−/−**^ (**g**–**i**) MNT-1 cells. Statistical significance was determined by two-way ANOVA followed by Bonferroni multiple comparison test relative to DMSO control (100 µM). (**j**) MNT-1 WT and TPC2^**−/−**^ cells treated with DMSO show significant difference in migration at 24, 48, and 72 h post treatment. (**k**) Wound closure of DMSO treated WT vs TPC2^**−/−**^ MNT-1 cells at 72 h post treatment. (**l**) Wound closure process pictured at 0 and 72 h post treatment in MNT-1 WT and TPC2^**−/−**^ cells treated with flavonoids or DMSO control. (**m**) MNT-1 WT (left) and TPC2^**−/−**^ (right) cells treated with 100 µM of NAR, 45 µM of MT-8, and 10 µM of UM-9 show significantly slower migration rates upon 72 h treatment compared to DMSO control (100 µM). (**n**) Invasion pictured at 24 h post treatment in MNT-1 WT and TPC2^**−/−**^ cells treated with flavonoids or DMSO control. (**o**) MNT-1 WT and TPC2^**−/−**^ cells treated with DMSO show significant difference in invasion at 24 h post treatment. (**p**) MNT-1 WT (left) and TPC2^**−/−**^ (right) cells treated with 100 µM of NAR, 45 µM of MT-8, and 10 µM of UM-9 show significantly slower invasion rates 24 h post treatment compared to DMSO control (100 µM). Statistical significance was determined by either two-way ANOVA followed by Bonferroni multiple comparison test (**j**), Student’s t-test (**k**, **o**), or one-way ANOVA followed by Bonferroni multiple comparison test (**m**, **p**). Shown are mean values ± SEM, (n = 3, each). *P < 0.05, **P < 0.01, ***P < 0.001, ****P < 0.0001.

An anti-cancer potential of flavonoids has long been claimed^15–17^. Proposed anticancer mechanisms for flavonoids are inhibition of proliferation, inflammation, invasion, metastasis, and activation of apoptosis^15^. We first tested the effect of vehicle (DMSO) on WT and TPC2^**−/−**^ MNT-1 cell proliferation (Fig. 5a) and confirmed highly significant assay windows between WT and TPC2^**−/−**^ MNT-1 cells at 48 and 72 h after treatment. Next, we assessed whether the effect of flavonoids, in particular of the hit compounds MT-8 and UM-9 as well as NAR on melanoma cell proliferation was mediated by TPC2. We found that MT-8 and UM-9 reduced proliferation of MNT-1 WT cells efficiently to the levels of TPC2^**−/−**^ MNT-1 cells while application of the compounds on TPC2^**−/−**^ MNT-1 cells showed no significant effect, indicating on-target activity of MT-8 and UM-9 (Fig. 5b–i). Likewise, NAR reduced proliferation in a TPC2-dependent manner but dose–response measurements revealed significant effects for NAR only at concentrations of > 80 µM (Fig. 5g) which is in agreement with the high IC_50_ measured in TPC2 inhibition experiments. Next, we assessed the effect of the compounds and DMSO control on migration of WT and TPC2^**−/−**^ MNT-1 cells. We confirmed the highly significant assay windows between WT and TPC2^**−/−**^ MNT-1 cells for migration at different time points (Fig. 5j–k). Application of MT-8, UM-9, and NAR reduced the migration efficiency of MNT-1 WT cells to TPC2^**−/−**^ values (Fig. 5l–m) while addition of the compounds to TPC2^**−/−**^ MNT-1 cells did not significantly reduce migration efficiency any further (Fig. 5l–m), again corroborating on-target effects of the compounds. Likewise, we assessed the effect of the compounds and DMSO control on invasion of WT and TPC2^**−/−**^ MNT-1 cells (Fig. 5n–p). Application of MT-8, UM-9, and NAR reduced the invasion efficiency of MNT-1 WT cells to TPC2^**−/−**^ values while addition of the compounds to TPC2^**−/−**^ MNT-1 cells did not significantly reduce invasion efficiency any further (Fig. 5n–p).

### MITF protein levels are reduced in TPC2 knockout melanoma cells while GSKβ levels are increased

**Figure 6:**
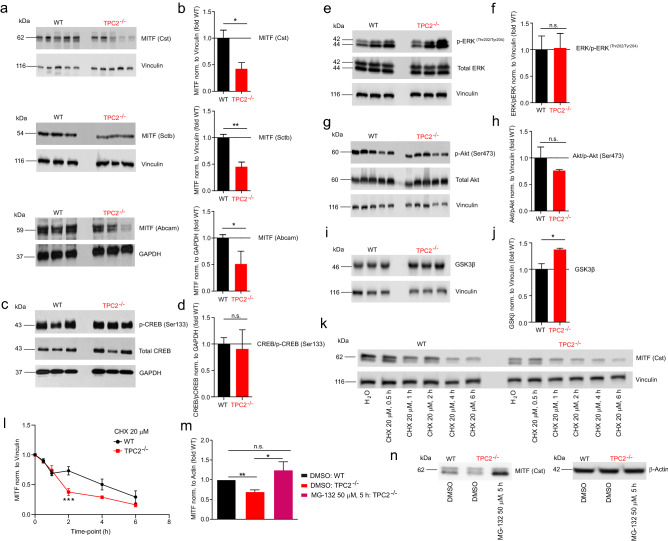
Effect of TPC2 knockout on MITF expression and expression of MITF regulator proteins using Western blot analysis and effect of protein synthesis and proteasome inhibition on MITF expression in WT and TPC2^**−/−**^ MNT-1 cells. Western blot experiments were performed with WT and TPC2^**−/−**^ MNT-1 cells as described in the Methods section. (**a**) and (**b**) WB experiments (**a**) and statistical analysis (**b**) showing MITF expression levels in WT and TPC2^**−/−**^ cells detected using three different anti-MITF antibodies (Cst = Cell Signaling Technology, Sctb = Santa Cruz Biotechnology). (**c**–**j**) WB experiments (**c**, **e**, **g**, **i**) and statistical analyses (**d**, **f**, **h**, **j**) showing expression levels of CREB/pCREB, ERK/pERK, Akt/pAkt and GSK3β in WT and TPC2^**−/−**^ cells detected using antibodies as described in the Methods section. Statistical significance was determined by Student’s t-test. Shown are mean values ± SEM, (n = 3, each). *P < 0.05, **P < 0.01. (**k**–**l**) WB experiments (**k**) and statistical analysis (**l**) showing the effect of cycloheximide (CHX) on MITF degradation in WT and TPC2^**−/−**^ MNT-1 cells. Statistical significance was determined by two-way ANOVA followed by Bonferroni multiple comparison test. Shown are mean values ± SEM, (n = 3, each). ***P < 0.001. (**m**) and (**n**) Rescue effect of the proteasome inhibitor MG-132 on MITF expression in TPC2^**−/−**^ MNT-1 cells. Statistical significance was determined by Student’s t-test. Shown are mean values ± SEM, (n = 3, each). *P < 0.05, **P < 0.01.

**Figure 7:**
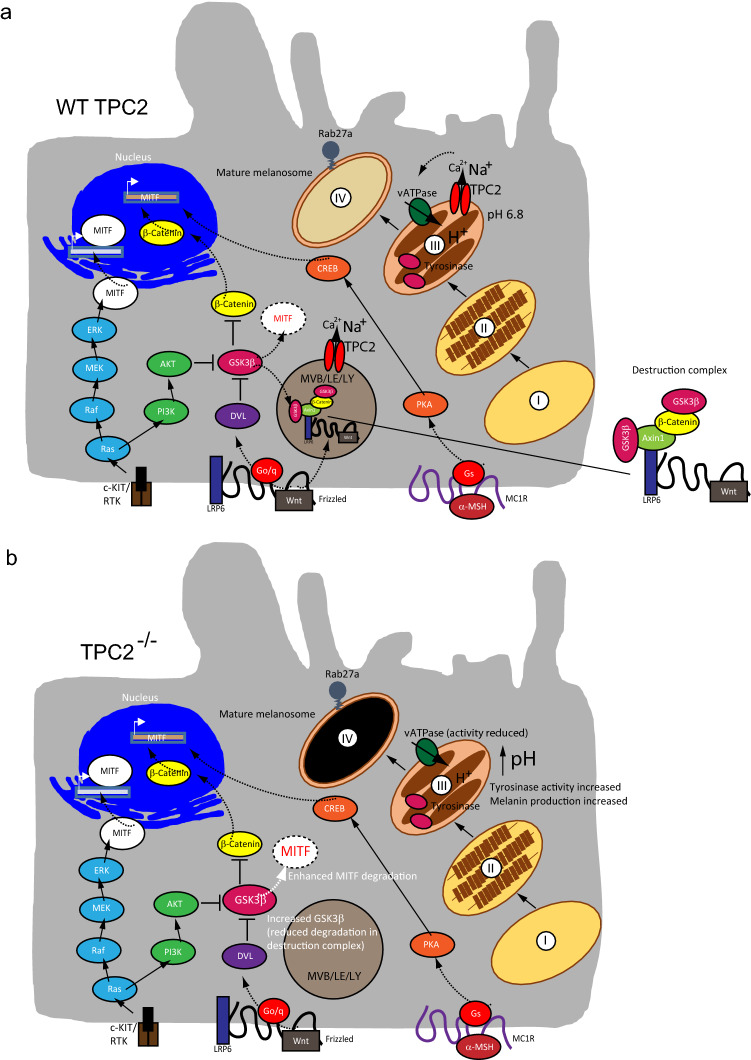
MITF signalling pathways and proposed effects in TPC2 knockout melanoma cells. (**a**) and (**b**) Cartoons showing MITF signalling pathways in WT (**a**) and TPC2^**−/−**^ cells (**b**). Several pathways regulate MITF expression: RAS/RAF/MEK/ERK, PI3K/AKT, Wnt/GSK3β/β-Catenin, and MSH/CREB signalling pathways. GSK3β is a negative regulator of MITF expression and promotes proteasomal degradation of MITF. GSK3β degradation in endolysosomes is enhanced by Wnt signalling. Tyrosinase activity depends on melanosomal pH and is regulated by TPC2 activity in melanosomes. Loss-of-function of TPC2 in endolysosomes and melanosomes results in increased GSK3β and decreased MITF protein levels as well as increased tyrosinase activity and melanin content.

MITF is a major regulator of melanoma proliferation and progression, and mutations in MITF are associated with Tietz albinism-deafness syndrome, Waardenburg syndrome type 2A, and melanoma development^18,19^. Several pathways are involved in the regulation of MITF such as the RAS/RAF/MEK/ERK pathway, the PI3K/AKT, and the Wnt/GSK3β/β-Catenin signalling pathways^20^. Melanin formation is also triggered by melanocyte-stimulating hormone (MSH), a peptide hormone encoded by the *proopiomelancortin* gene (POMC). MSH binding to MC1R results in the induction of MITF via CREB (cAMP response element-binding protein)^20^. We performed Western blot experiments to assess protein levels of MITF and several key proteins involved in the regulation of MITF expression. We found that in TPC2^**−/−**^ MNT-1 cells MITF protein levels were strongly decreased compared to WT cells. We confirmed this result by using three different antibodies against MITF (Fig. 6a,b and Fig. S4). We next assessed the expression levels of CREB, ERK, Akt, and GSK3β (Fig. 6c–j, Fig. S4, and Fig. 7). While ERK, Akt, and CREB showed no significant differences, the expression of GSK3β was significantly increased. GSK3β is a negative regulator of MITF expression and can target MITF for proteasomal degradation. Activation of Wnt signalling can prevent this process by increasing the endolysosomal destruction of GSK3β^21^. An increased level of GSK3β suggests reduced degradation of the GSK3β containing destruction complexes in endolysosomes, resulting in increased GSK3β-dependent MITF degradation.

To further corroborate this hypothesis we assessed the MITF protein stability in WT and TPC2^**−/−**^ MNT-1 cells by using cycloheximide (CHX). CHX is an inhibitor of protein synthesis and can be used to determine the longevity of proteins^22^. In Western blot experiments we found an increased sensitivity of TPC2^**−/−**^ compared to WT MNT-1 cells to CHX treatment, resulting in a faster and significantly stronger degradation of MITF in TPC2^**−/−**^ MNT-1 cells (Fig. 6k–l and Fig. S4). To demonstrate that the differences in degradation of MITF were dependent on proteasomal activity we used the proteasome inhibitor MG-132. MG-132 was found to reestablish WT MITF levels within 5 h after treatment to TPC2^**−/−**^ MNT-1 cells (Fig. 6m–n), indicative of proteasomal degradation causing the reduction in MITF levels.

## Discussion

We show here a TPC2-dependent inverse correlation between melanin generation and melanoma proliferation, migration, and invasion by using both genetic and pharmacological approaches. While genetic ablation or pharmacological inhibition increase melanin production in a TPC2-dependent manner, proliferation, migration, and invasion of melanoma cells are reduced. We further found that the flavonoids MT-8 (pratensein) and UM-9 (duartin) efficiently reduce melanoma cell proliferation, migration, and invasion in a TPC2-dependent manner, thus providing a molecular rationale for previously suggested anti-cancer effects of flavonoids^15–17^. Besides MT-8 and UM-9, the related compound MT-16 which corresponds to NAR was confirmed as a TPC2 inhibitor. NAR had been demonstrated before to impair VEGF-induced vessel formation^9,10^ in a TPC2 dependent manner. Flavonoids thus emerge as anti-cancer drugs acting through the endolysosomal/melanosomal cation channel TPC2.

Melanin content and the development of melanoma have previously been suggested to be correlated^12^ and it is well established that melanin is one of the major protective factors against UV radiation mediated DNA damage that results in melanoma development. TPC2 now emerges as a critical regulator of both melanin generation and melanoma proliferation, migration, and invasion in human melanoma cells due to its dual functions in melanosomes and endolysosomes.

Through regulation of genes related to invasiveness, migration and metastasis MITF can promote melanoma progression^19^. We found here that knockout of TPC2 results in a strong decrease in MITF protein abundance, suggesting that the reduction in MITF levels is likely causative for the observed reduced effects on proliferation/migration/invasion in TPC2^**−/−**^ MNT-1 cells. Wnt signalling is known to stabilize MITF protein levels in melanoma cells. Ploper et al. (2015) have shown that Wnt signalling can also regulate MITF at the protein degradation level, underscoring the importance of misregulated endolysosomal biogenesis and trafficking in Wnt signalling and cancer^21^. Wnt inhibits GSK3β and promotes sequestration of destruction complexes containing GSK3β into endosomes and MVBs, thus stabilizing MITF protein levels (Fig. 7). In the absence of Wnt and Wnt signalling GSK3β phosphorylates MITF, targeting MITF for proteasomal degradation. In TPC2^**−/−**^ cells endolysosomal trafficking and degradation are impacted as shown before for, e.g. LDL, EGF/EGFR, or PDGFR trafficking and degradation^23–25^, suggesting a possible impact on the sequestration and degradation of the destruction complex containing GSK3β. Consequently, we found increased GSK3β protein levels and an increased proteasomal degradation of MITF. Of note, flavonoids have been reported before to affect MITF expression through interference with Wnt signalling. Thus, e.g. Syed et al. (2011) showed that human melanoma cell growth inhibition by flavonoids was associated with disruption of Wnt signalling and decreased MITF levels^26^. Such data further support a connection between flavonoids, TPC2, Wnt signalling and MITF expression.

A role of TPC2 and TPC2 variation in melanoma development is also supported by genome-wide association studies. Thus, Kosiniak-Kamysz et al. (2014) examined 33 candidate polymorphisms located in 11 pigmentation genes and the vitamin D receptor (VDR) gene in a population of 130 cutaneous melanoma patients and 707 healthy controls^27^. In the final multivariate analysis with genetic interactions included and after adjustment for age and skin colour, five epistatic effects remained significant, i.e. interactions between MC1R and TYR, SLC45A2 and VDR, HERC2 and VDR, OCA2 and TPC2. The identified TPC2 variant rs3829241 (P = 0.007) is one (TPC2^G734^^E^) of the two variants shown to be GOF variants^6^ and to be associated with blond hair color in humans^5^.

Taken together, our data provide strong evidence not only for a role of TPC2 in cancer proliferation, migration, and invasion in general but specifically a twofold role for TPC2 in melanoma development by affecting on the one hand MITF protein abundance and on the other hand melanin production independently of MITF through direct interference with tyrosinase activity in melanosomes. Thus, melanoma cell proliferation, migration, and invasion are inversely correlated with TPC2-dependent melanin production as reduction of TPC2 expression increases melanin content but decreases proliferation/migration/invasion. This is possible due to independent mechanisms: via regulation of MITF protein levels through interference with endolysosomal activity of TPC2 and endolysosomal GSK3β degradation on the one hand and on the other hand via regulation of tyrosinase activity in melanosomes which likewise express TPC2^28^. In a very recent study, D’Amore et al. (2020) have also investigated the role of TPC2, but in a model of human amelanotic melanoma: CHL1. In CHL1 cells, TPC2 was surprisingly found to increase the metastatic traits of this amelanotic melanoma cell line by a mechanism involving store-operated calcium entry and the Hippo signalling pathway that negatively regulates YAP/TAZ activity. Clearly, these differences between amelanotic melanoma cells (CHL1) on the one hand and highly pigmented melanoma cells (MNT-1) but also a range of other cancer cells (e.g., HUH7, T24, 4T1)^7^ on the other hand regarding TPC2 need to be further elucidated in future studies.

## Materials and methods

### Endolysosomal patch-clamp experiments

Endolysosomal patch-clamp experiments were performed as previously described^6,14,23,25,29,30^. In brief, for whole-LE/LY manual patch-clamp recordings, cells were treated with 1 μM vacuolin (HEK293 cells: overnight) in an incubator at 37 °C with 5% CO_2_. Compound was washed out before patch-clamp experimentation. Currents were recorded using an EPC-10 patch-clamp amplifier (HEKA, Lambrecht, Germany) and PatchMaster acquisition software (HEKA). Data were digitized at 40 kHz and filtered at 2.8 kHz. Fast and slow capacitive transients were cancelled by the compensation circuit of the EPC-10 amplifier. All recordings were obtained at room temperature and were analyzed using PatchMaster acquisition software (HEKA) and OriginPro 6.1 (OriginLab). Recording glass pipettes were polished and had a resistance of 4–8 MΩ. For all experiments, salt-agar bridges were used to connect the reference Ag–AgCl wire to the bath solution to minimize voltage offsets. Liquid junction potential was corrected. For the application of small molecules, compounds were added directly to the patched endolysosomes to either evoke or inhibit the current. The cytoplasmic solution was completely exchanged by cytoplasmic solution containing compound. The current amplitudes at −100 mV were extracted from individual ramp current recordings. Unless otherwise stated, cytoplasmic solution contained 140 mM K-MSA, 5 mM KOH, 4 mM NaCl, 0.39 mM CaCl_2_, 1 mM EGTA and 10 mM HEPES (pH was adjusted with KOH to 7.2). Luminal solution contained 140 mM Na-MSA, 5 mM K-MSA, 2 mM Ca-MSA 2 mM, 1 mM CaCl_2_, 10 mM HEPES and 10 mM MES (pH was adjusted with methanesulfonic acid to 4.6). In all experiments, 500-ms voltage ramps from − 100 to + 100 mV were applied every 5 s. All statistical analysis was completed using OriginPro9.0 and GraphPadPrism software.

### Cell culture

HEK293 cells stably expressing hTPC2-YFP or hTRPML1-YFP were used for patch-clamp experiments. Cells were maintained in DMEM supplemented with 10% FBS, 100 U penicillin/mL, and 100 μg streptomycin/mL. Cells were plated on glass cover slips 24–48 h before experimentation. Cells were transiently transfected with Turbofect (Fermentas) according to the manufacturer’s protocols and used, e.g. for confocal imaging or patch-clamp experiments 24–48 h after transfection. Cells were treated with compounds at 37 °C and 5% CO_2_. MNT-1 WT and TPC2^**−/−**^ KO cell lines were grown in high glucose DMEM, supplemented with 20% FBS, 10% AIM-V, 1% sodium pyruvate (Thermo Fisher), and 1% penicillin–streptomycin (Sigma-Aldrich). B16F10 cells were grown in high glucose DMEM, supplemented with 10% FBS (Thermo Fisher), 1% L-glutamin, and 1% penicillin–streptomycin (Sigma-Aldrich). Cell lines were maintained at 37 °C in a 5% CO_2_ incubator.

### Melanin screening in B16F10 mouse melanoma cells

Melanin content determination was performed as described previously with some modifications^31^. In brief, B16F10 cells at density of 5 × 10^3^ cells/well in 96-well plate were cultured and incubated with various plant extracts or flavonoids at a concentration of 20 µg/ml or 20 µM, respectively, for 4–5 days. Melanin content was measured using a microplate reader (Anthros, Durham, NC, USA) and calculated based on the OD ratio between treated and untreated cells.

### Melanin content and tyrosinase activity assays

MNT-1 WT and TPC2^**−/−**^ KO cell lines were grown as described in the cell culture section. After reaching 80–90% confluency, cells were subcultured (every 2–3 days). Forskolin (Sigma-Aldrich Cas Nr. 66,575,299) was used as positive control and 4-Butyl-resorcinol (TYR-inh., Sigma-Aldrich, Cas Nr.18979-61-8) as negative control. For experiments, cells were plated in 6-well plates with 200,000 cells per well. Cells were incubated for 72 h at 37 °C and 5% CO_2_. After removing cell culture media, cells were washed in DPBS twice, then cells were collected using a cell scraper. Cells were centrifuged at 3000 rpm for 5 min. Pellets were lysed with RIPA buffer, supplemented with 1% protease inhibitor cocktail (Sigma-Aldrich) and 1% phosphatase inhibitor (Sigma-Aldrich) at 4 °C (on ice) for 45 min. Cells were centrifuged at 12.000 rpm for 15 min (4 °C), supernatant was subsequently removed and protein content determined using a protein dye reagent assay (Bio-Rad; protein standard curve (BSA) 0, 1, 3, 5, 8, 10, 12, 15 μg/mL). Cell pellets were dissolved in 250 μL 1 N NaOH/10% DMSO and incubated at 80 °C for 2 h. After centrifugation at 12.000 rpm for 10 min, supernatants were removed to a 96-well plate. Absorbance was measured (in triplicates, each) at 405 nm using a microplate reader (Tecan, Infinite M200 PRO). Melanin content was normalized to total protein content.

To measure tyrosinase activity 100 μg protein from the supernatant after RIPA lysis were transferred into a 96-well plate and 50 μL of 15 mM L-DOPA (Sigma) were added (total volume was adjusted to 100 μL using PBS, pH 6.8 (adjusted with 1 N HCl)). After 30 min incubation at 37 °C, dopachrome formation was determined by measuring the absorbance at 475 nm using a microplate reader (Tecan, Infinite M200 PRO). Tyrosinase activity (%) was calculated as follows: OD475 (sample) × 100 / OD475 (control).

### Cell proliferation assay

Cell proliferation assay was performed in 96-well, flat-bottom microtiter plates (Sarstedt), in triplicates, and at a 5 × 10^3^ cell density per well. Cells were seeded overnight, including cells measured as day zero control. Proliferation rate was assessed by incubation with CellTiter-Blue (Ctb, Promega, Mannheim, Germany) reagent for 3 h. Fluorescence was measured using a microplate reader at 560Ex/600Em (Tecan, Infinite M200 PRO).

### Wound healing/migration assay

Wound healing assay was performed using 12-well plates (Sarstedt) at a density of 120,000 cells/well. Cells were incubated overnight, and a scratch was performed using a yellow pipet tip. Pictures were taken at 0, 24, 48, and 72 h with an inverted microscope (Leica DM IL LED) and using a microscope camera (Leica DFC 3000 G). The wounded cell area was quantified using ImageJ 1.52a software and was subtracted from 0 h values.

### Invasion assay

Transwell chambers in 24-well permeable support plates (Corning, #3421) were coated with Corning Matrigel basement membrane matrix (Corning, #354234) for 1.5 h. A total of 3 × 10^4^ MNT-1 cells were seeded on top of the chambers in serum-free medium, and direct stimulation with compounds was performed. The lower compartment contained the chemotactic gradient, medium with 10% FBS. Cells were allowed to migrate for 24 h, and were then fixed and stained with crystal violet containing methanol. Non-invaded cells were removed with Q-tips and pictures were taken of the bottom side of the membrane using an inverted microscope (Olympus CKX41) and an Olympus SC50 camera (Olympus). The number of invaded cells was quantified using ImageJ 1.52a software.

### Western blotting

Western blot experiments were performed as described previously^32^. Briefly, cells were washed twice with 1 × PBS and pellets were collected. Total cell lysates were obtained by solubilizing in TRIS HCl 10 mM pH 8.0 and 0.2% SDS supplemented with protease and phosphatase inhibitors (Sigma). Protein concentrations were quantified via Bradford assay. Proteins were separated via a 10% sodium dodecyl sulphate polyacrylamide gel electrophoresis (SDS-PAGE; BioRad) and transferred to polyvinylidene difluoride (PVDF; BioRad) membranes. Membranes were blocked with 5% bovine serum albumin (Sigma) or milk diluted in Tris Buffered Saline supplemented with 0.5% Tween-20 (TBS-T) for 1 h at room temperature (RT), then incubated with primary antibody at 4 °C overnight. Then, membranes were washed with TBS-T and incubated with horseradish peroxidase (HRP) conjugated anti-mouse or anti-rabbit secondary antibody (Cell Signaling Technology) at RT for 1 h. Membranes were then washed and developed by incubation with Immobilon Crescendo Western HRP substrate (Merck) and by using an Odyssey imaging system (LI-COR Biosciences). Quantification was carried out using unsaturated images on ImageJ 1.52a software. The blots were cropped prior to hybridisation with antibodies against vinculin, GAPDH, or actin. The following antibodies were used: Phospho-p44/42 MAPK (Erk1/2) (Thr202/Tyr204) (Cell Signaling Technology, 1:1000, cat. #9106), p44/42 MAPK (Erk1/2) (Thr202/Tyr204) (Cell Signaling Technology, 1:1000, cat. #9102), Phospho-Akt (Ser473) (Cell Signaling Technology, 1:1000, cat. #4058), Akt (Cell Signaling Technology, 1:1000, cat. #9272), MITF (Santa Cruz Biotechnology, 1:1000, cat. #Sc-71588), MITF (Cell Signaling Technology, 1:1000, cat. #97800), MITF (Abcam, 1:1000, cat. #ab12039), GSK-3β (Cell Signaling Technology, 1:1000, cat. #9832), CREB and pCREB (Cell Signaling Technology, 1:1000, cat. #9197S and #9198S), ß-Actin (Santa Cruz Biotechnology, 1:1000, cat. #Sc-47778), Vinculin (Cell Signaling Technology, 1:1000, cat. #4650), GAPDH (Cell Signaling Technology, 1:1000, cat. #5174S), Anti-Mouse (Cell Signaling Technology, 1:10,000, cat. #7076), and Anti-Rabbit (Cell Signaling Technology, 1:10,000, cat. #7074).

### RNA isolation and quantitative PCR

Total RNA was isolated from the cells using the RNeasy Mini Kit (Qiagen). Reverse Transcription was performed using the Revert First Strand cDNA Synthesis Kit (Thermo Fisher). Real-time quantitative Reverse Transcription PCR (qPCR) was performed in triplicates for each sample using the LightCycler 480 SYBR Green I Master and using the LightCycler 480 II machine (Roche Life Science), following the recommended parameters. HPRT was used as the housekeeping gene. The following human primer sets were used: Tyrosinase primers set A: fw: 5′-GTCTGTAGCCGATTGGAGGA -3′; rev: 5′- TGGGGTTCTGGATTTGTCAT -3′. Tyrosinase primers set B: fw: 5′-TGACAG TATTTTTGAGCAGTGG -3′; rev: 5′- GGTGCATTGGCTTCTGGATA-3'.

### Plant material

Commercially available heartwood of *Dalbergia parviflora* was purchased from “Chao Krom Poe” herbal medicine dispensary in Bangkok in 2004. The samples were identified as wild *Dalbergia parviflora* at Princess Sirindhorn Wildlife Sanctuary, known as “Pa Phru To Daeng” which is a peat swamp forest in Mueang Narathiwat, Tak Bai, Su-ngai Kolok, and Su-ngai Padi districts of Narathiwat Province in Southern Thailand (06° 04′ 33.8″ N, 101° 57′ 49.3″ E). Data collection in the area was carried out with the authorization and guidelines of the National Research Council of Thailand (NRCT), and complied with the IUCN Policy Statement on Research Involving Species at Risk of Extinction and the Conservation (1989) and the Convention on International Trade in Endangered Species of Wild Fauns and Flora (CITES, 1975). The plant was identified by Dr. Chawalit Niyomdham of the Forest Herbarium, National Park, Wildlife and Plant Conservation Department, Bangkok, Thailand. Its voucher specimen (number 68143)^33,34^ was deposited at The Forest Herbarium, Bangkok, Thailand.

### Extraction and isolation of flavonoids

The dried heartwood of *D. parviflora* (2 kg) was extracted three times with MeOH (3 × 20 L) at room temperature. The extracts were combined and concentrated under reduced pressure at 60 °C to yield 910 g of a viscous mass. A part of this concentrated extract (150 g) was chromatographed on a silica gel column (12 × 40 cm) and fractionated using chloroform-MeOH (98:2, 96:4, 94:6, 90:10, 15 L each). Fractions of 500 mL were collected and pooled by TLC analysis to yield a total of 26 combined fractions. Purification of these fractions as reported previously^33,34^ gave various flavonoid compounds as summarized in Fig. S1. Purification of fraction 14 (8.9 g) using HPLC on a Develosil- Lop-ODS column (5 × 100 cm, flow rate, 45 mL/min with detection at 205 nm), with MeCN-H_2_O (30:70) as the eluent gave MT-8 (pratensein) (715 mg) (*t*_R_ = 220 min). Purification of fraction 6 (3.1 g) using HPLC on a Develosil-Lop-ODS column (5 × 100 cm, flow rate: 45 mL/min with detection at 205 nm), with MeCN-H_2_O (32:68) as the eluent, gave UM-9 (duartin) (39 mg) (*t*_R_ = 240 min). Both compounds were identified by comparison of their spectroscopic data with published values^35,36^.

### NMR analytical data

NMR spectra were measured on an JEOL alpha 400 (^1^H-NMR: 400 MHz, ^13^C-NMR: 100.4 MHz) spectrometer^33,34^. NMR-Spectra were measured in deuterated solvents and chemical shifts are reported in δ (ppm) relative to the internal standard tetramethylsilane (TMS) or the solvent peak at 35 °C, respectively. *J* values are given in hertz. Multiplicities are abbreviated as follows: s = singlet, d = doublet, t = triplet, q = quartet, m = multiplet. Signal assignments were carried out based on ^1^H, ^13^C, HMBC, HMQC and COSY spectra. Inverse-detected heteronuclear correlations were measured using HMQC (optimized for ^1^*J*_C-H_ = 145 Hz) and HMBC (optimized for ^3^*J*_C-H_ = 8 Hz) pulse sequences with a pulsed field gradient. FABMS spectra were obtained on a JEOL JMS-700 using a *m*-nitrobenzyl alcohol matrix. Optical rotation was measured on a JASCO DIP-360 digital polarimeter. Column chromatography (CC) was performed with powdered silica gel (Kieselgel 60, 230–400 mesh, Merck KGaA, Darmstadt, Germany) and styrene–divinylbenzene (Diaion HP-20, 250–800 µm particle size, Mitsubishi Chemical Co., Ltd.). Precoated glass plates of silica gel (Kieselgel 60, F254, Merck Co., Ltd., Japan) and RP-18 (F254S, Merck KGaA) were used for TLC analysis. The TLC spots were visualized under UV light at a wavelength of 254 nm and sprayed with dilute H_2_SO_4_, followed by heating. HPLC separation was mainly performed with a JASCO model 887-PU pump, and isolates were detected by an 875-UV variable-wavelength detector. Reversed-phase columns for preparative separations (Develosil Lop ODS column, 10—20 µm, 5 × 50 × 2 cm; Nomura Chemical Co. Ltd., Aichi, Japan; flow rate 45 mL/min with detection at 205 nm) and semi-preparative separations (Capcell Pak ODS, 5 µm, 2 × 25 cm, Shiseido Fine Chemiacls Co. Ltd, Tokyo, Japan; flow rate 9 mL/min with detection at 205 nm) were used. MT-8 (pratensein): Amorphous powder; ^1^H-NMR (400 MHz, (CD_3_)_2_CO) δ (ppm) = 13.03 (s, 1H, 5-H), 8.18 (s, 1H, 2-H), 7.13 (d, *J* = 2 Hz, 1H, 2′-H), 7.04 (dd, *J* = 9, 2 Hz, 1H, 6′-H), 6.99 (d, *J* = 9 Hz, 1H, 5′-H), 6.41 (d, *J* = 2 Hz, 1H, 8-H), 6.28 (d, *J* = 2 Hz, 1H, 6-H), 3.87 (s, 3H, 4′-OCH_3_). ^13^C-NMR (100.4 MHz, (CD_3_)_2_CO) δ (ppm) = 181.6 (C-4), 165.0 (C-7), 164.0 (C-5), 159.1 (C-9), 154.5 (C-2), 165.0 (C-7), 148.6 (C-4′), 147.3 (C-3′), 125.0 (C-1′), 121.3 (C-6′), 124.0 (C-3), 112.3 (C-5′), 106.3 (C-10), 99.9 (C-6), 94.5 (C-8), 56.4 (C-4′ OCH_3_). FABMS *m/z* 323 [MNa] + (calcd for C_16_H_12_O_6_Na). UM-9 (duartin): morphous powder; ^1^H-NMR (400 MHz, (CD_3_)_2_CO) δ (ppm) = 6.70 (d, *J* = 9 Hz, 1H, 5′-H), 6.65 (d, *J* = 9 Hz, 1H, 6′-H), 6.64 (d, *J* = 9 Hz, 1H, 5-H), 6.40 (d, *J* = 9 Hz, 1H, 6-H), 4.29 (ddd, *J* = 10, 3, 2 Hz, 1H, 2 eq-H), 3.96 (t, *J* = 10 Hz, 1H, 2ax-H), 3.47 (dddd, *J* = 11, 10, 5, 3 Hz, 1H, 3-H), 2.91 (dd, *J* = 16, 11 Hz, 1H, 4ax-H), 3.47 (ddd, *J* = 16, 5, 2 Hz, 1H, 4 eq-H), 3.87 (s, 3H, 2′-OCH_3_) , 3.81 (s, 3H, 4′-OCH_3_) , 3.77 (s, 3H, 8-OCH_3_). C-NMR (100.4 MHz, (CD_3_)_2_CO) δ (ppm) = 149.4 (C-7), 148.5 (C-9), 148.3 (C-4′), 146.5 (C-2′), 140.2 (C-3′), 136.6 (C-8), 128.0 (C-1′), 124.5 (C-6), 117.2 (C-6′), 115.4 (C-10), 108.4 (C-6), 107.9 (C-5′), 70.8 (C-2), 32.5 (C-2), 32.1 (C-3), 60.7 (C-8 OCH_3_), 60.5 (C-2′ OCH_3_), 56.4 (C-4′ OCH_3_). [α]_D_ + 15.4° (*c* 1.0, CHCl_3_). FABMS *m/z* 355 [MNa] + (calcd for C_18_H_20_O_6_Na).

## Statistical analysis

Details of statistical analyses and n values are provided in the “Materials and Methods” or the Figures or Figure legends. Statistical analyses were carried out using Origin 8 and GraphPad Prism 8. All error bars are depicted as mean ± SEM. Statistical significance is denoted on Figures as outlined in the legends.

## Supplementary Information

Supplementary Information.

## Data Availability

All data generated or analyzed during this study are included in this published article and its additional files.
