# Supplementary material for: Flavonoids increase melanin production and reduce proliferation, migration and invasion of melanoma cells by blocking endolysosomal/melanosomal TPC2
Source: Sci Rep. 2021 Apr 19;11:8515. doi: 10.1038/s41598-021-88196-6 (PMC8055690; doi:10.1038/s41598-021-88196-6)

Supplementary Information

Flavonoids increase melanin production and reduce proliferation, migration and invasion of melanoma cells by blocking endolysosomal/melanosomal TPC2

Ponsawan Netcharoensirisuk[1,2#], Carla Abrahamian[1#], Rachel Tang[1#], Cheng-Chang Chen[3], Anna Scotto Rosato[1], Wyatt Beyers[4], Yu-Kai Chao[1], Antonio Filippini[5], Santiago Di Pietro[4], Karin Bartel[3], Martin Biel[3], Angelika M. Vollmar[3], Kaoru Umehara[6], Wanchai De-Eknamkul[2*], Christian Grimm[1*]

Fig. S1. Western blots of tyrosinase, Dct (dopachrome tautomerase, TYRP2), Rab27a, and PMEL (premelanosome protein) in WT and TPC2$^{-/-}$ MNT-1 cells. Western blot experiments were performed with WT and TPC2$^{-/-}$ MNT-1 cells as described in the Methods section. (a-f) Representative WB experiments (a, c, e) and statistical analysis (b, d, f) showing tyrosinase, Dct and PMEL expression levels in WT and TPC2$^{-/-}$ cells detected using the following primary and secondary antibodies: Mouse anti tyrosinase (Cat No. SC20035, Santa Cruz Biotechnology, 1:500 or 1:1000), rabbit anti Dct (Abcam Cat No. ab74073, 1:2000), mouse anti Rab27a (Cat No. SC81914, Santa Cruz Biotechnology, 1:1000), mouse anti pmel17 (Cat No. SC377325, Santa Cruz Biotechnology, 1:1000), rabbit anti GAPDH (Cat No. 2118S, Cell Signaling Technology, 1:1000), and mouse anti ß-Actin (Protein Tech, 1:40000, Cat No. 66009-1-Ig). Secondary antibodies: anti-rabbit IgG HRP-Linked (Cat No. 7074S, Cell Signaling Technology, 1:1000), anti-mouse IgG HRP-Linked (Cat No. 7076S, Cell Signaling Technology, 1:1000), and anti-mouse (Sigma, 1:10000, cat. # GENA931). (g) qRT-PCR results using primer sets A and B as described in the Methods section to determine tyrosinase transcript levels in WT and TPC2-/- MNT-1 cells. Statistical significance was determined by Student's t-test. Shown are mean values ± SEM, (n = 3, each). *P<0.05, ***P<0.001.

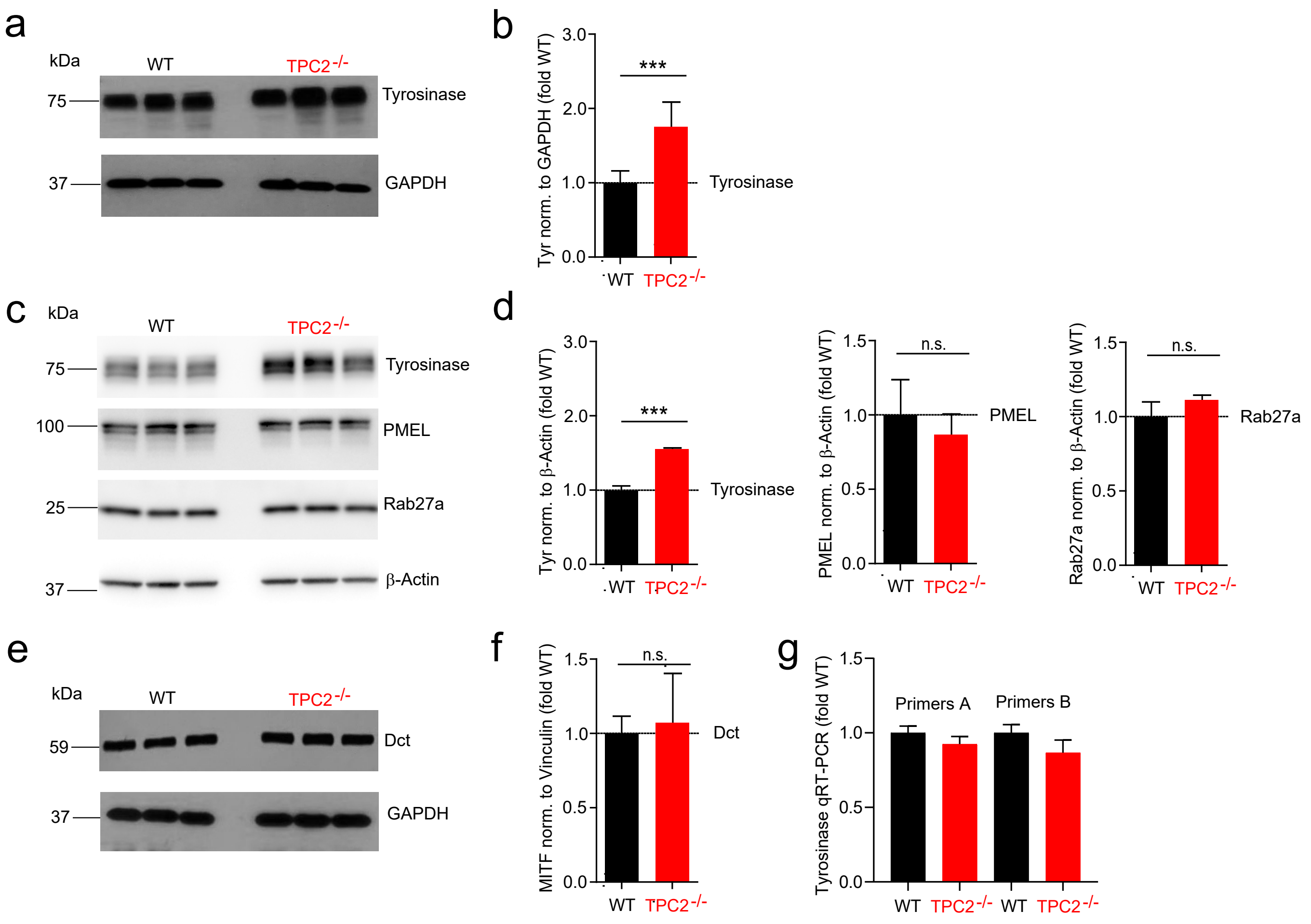
a
kDa
WT
TPC2-/-
75
Tyrosinase
37
GAPDH
b
Tyr norm. to GAPDH (fold WT)
3.0
2.0
1.0
0.0
***
Tyrosinase
WT
TPC2-/-
c
kDa
WT
TPC2-/-
75
Tyrosinase
100
PMEL
25
Rab27a
37
β-Actin
d
Tyr norm. to β-Actin (fold WT)
***
Tyrosinase
PMEL norm. to β-Actin (fold WT)
n.s.
PMEL
Rab27a norm. to β-Actin (fold WT)
n.s.
Rab27a
1.5
1.0
0.5
0.0
WT
TPC2-/-
e
kDa
WT
TPC2-/-
59
Dct
37
GAPDH
f
MITF norm. to Vinculin (fold WT)
n.s.
Dct
WT
TPC2-/-
g
Tyrosinase qRT-PCR (fold WT)
Primers A
Primers B
WT
TPC2-/-
WT
TPC2-/-

Fig. S2. Chemical structures of isolated flavonoids. All flavonoids used in the melanin content screens in B16F10 mouse melanoma and human MNT-1 melanoma cells are shown.

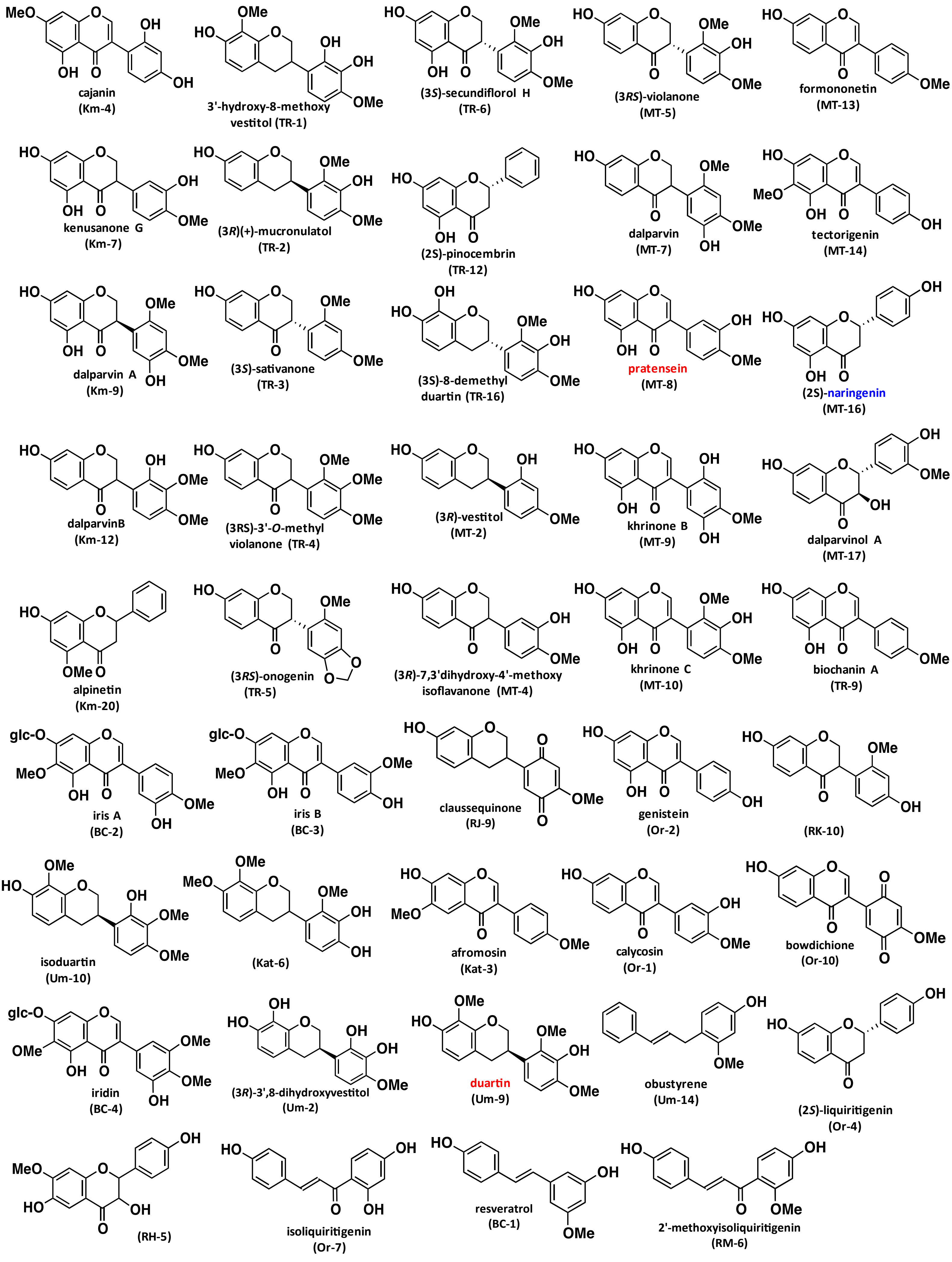
cajanin
(Km-4)
3'-hydroxy-8-methoxy vestitol (TR-1)
(3S)-secundiflorol H
(TR-6)
(3RS)-violanone
(MT-5)
formononetin
(MT-13)
kenusanone G
(Km-7)
(3R)(+)-mucronulatol
(TR-2)
(2S)-pinocembrin
(TR-12)
dalparvin
(MT-7)
tectorigenin
(MT-14)
dalparvin A
(Km-9)
(3S)-sativanone
(TR-3)
(3S)-8-demethyl duartin (TR-16)
pratensein
(MT-8)
(2S)-naringenin
(MT-16)
dalparvinB
(Km-12)
(3RS)-3'-O-methyl violanone (TR-4)
(3R)-vestitol
(MT-2)
khrinone B
(MT-9)
dalparvinol A
(MT-17)
alpinetin
(Km-20)
(3RS)-onogenin
(TR-5)
(3R)-7,3'dihydroxy-4'-methoxy isoflavanone (MT-4)
khrinone C
(MT-10)
biochanin A
(TR-9)
iris A
(BC-2)
iris B
(BC-3)
claussequinone
(RJ-9)
genistein
(Or-2)
(RK-10)
isoduartin
(Um-10)
(Kat-6)
afromosin
(Kat-3)
calycosin
(Or-1)
bowdichione
(Or-10)
iridin
(BC-4)
(3R)-3',8-dihydroxyvestitol
(Um-2)
duartin
(Um-9)
obustyrene
(Um-14)
(2S)-liquiritigenin
(Or-4)
(RH-5)
isoliquiritigenin
(Or-7)
resveratrol
(BC-1)
2'-methoxyisoliquiritigenin
(RM-6)

Fig S3. Concentration and time dependent effects of flavonoids on melanin content and tyrosinase activity in B16F10 cells. (a) and (b) Examples of experiments as quantified in Figure 1b. Cells were cultured in 96 well plates for 4-5 days until melanin content was assessed using a microplate reader at 405 nm. (c) and (d) Quantification of the time and concentration dependent effects of MT-8 on tyrosinase activity (c) and melanin content (d). (e) and (f) Quantification of the time and concentration dependent effects of UM-9 on tyrosinase activity (e) and melanin content (f). (g-i) Melanin content in WT and $TPC2^{-/-}$ MNT1 cells after treatment with different concentrations of MT-8, UM-9, and NAR. Statistical significance was determined by two-way ANOVA followed by Bonferroni multiple comparisons test. Shown are mean values ± SEM, (n = 3, each). $^{*}P<0.05$, $^{**}P<0.01$, $^{***}P<0.001$.

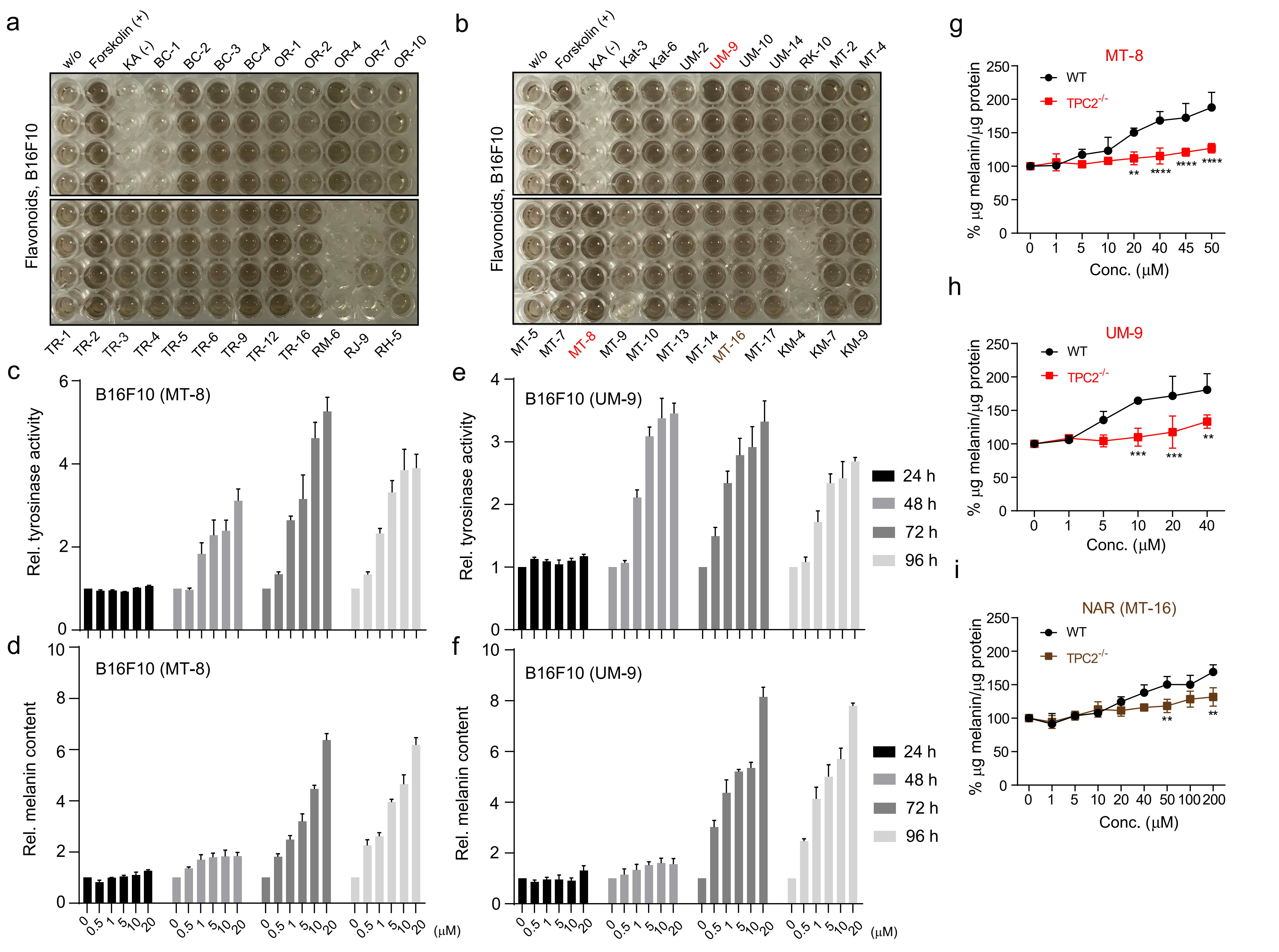

a
w/o
Forskolin (+)
KA (-)
BC-1
BC-2
BC-3
BC-4
OR-1
OR-2
OR-4
OR-7
OR-10
Flavonoids, B16F10
TR-1
TR-2
TR-3
TR-4
TR-5
TR-6
TR-9
TR-12
TR-16
RM-6
RJ-9
RH-5
b
w/o
Forskolin (+)
KA (-)
Kat-3
Kat-6
UM-2
UM-9
UM-10
UM-14
RK-10
MT-2
MT-4
Flavonoids, B16F10
MT-5
MT-7
MT-8
MT-9
MT-10
MT-13
MT-14
MT-16
MT-17
KM-4
KM-7
KM-9
c
B16F10 (MT-8)
Rel. tyrosinase activity
d
B16F10 (MT-8)
Rel. melanin content
e
B16F10 (UM-9)
Rel. tyrosinase activity
24 h
48 h
72 h
96 h
f
B16F10 (UM-9)
Rel. melanin content
24 h
48 h
72 h
96 h
(μM)
g
MT-8
WT
TPC2-/-
% μg melanin/μg protein
Conc. (μM)
h
UM-9
WT
TPC2-/-
% μg melanin/μg protein
Conc. (μM)
i
NAR (MT-16)
WT
TPC2-/-
% μg melanin/μg protein
Conc. (μM)

Figure S4. Whole western blots of proteins as shown in Figure 6 and Figure S1.

Fig.6a upper panel

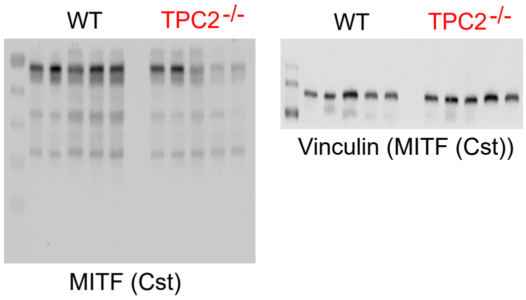

Fig.6a middle panel

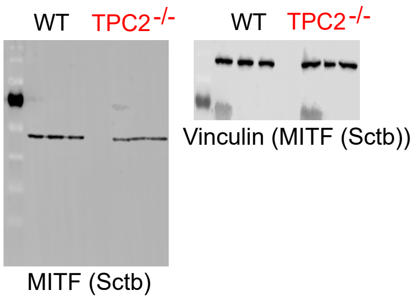

Fig.6a lower panel

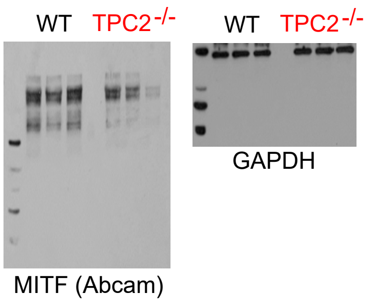

Fig.6c

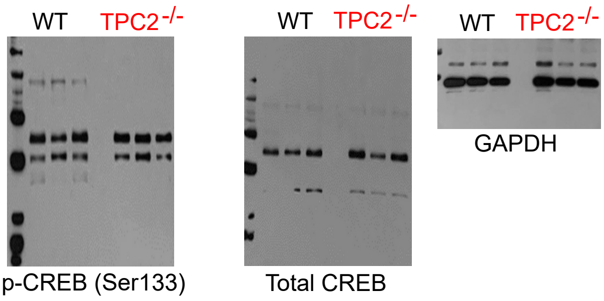

Fig.6e

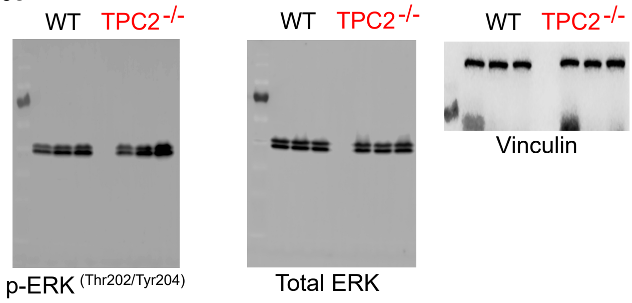

Fig.6g

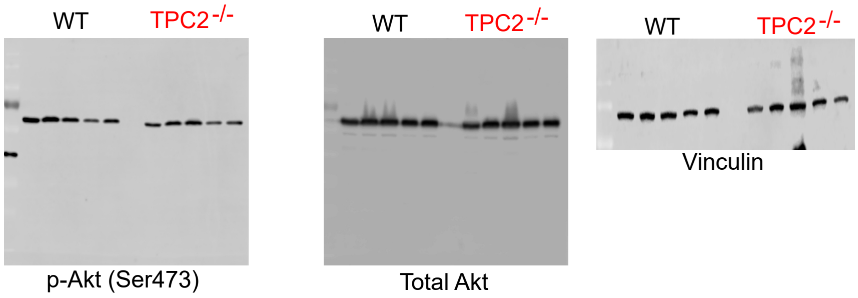

Fig.6i

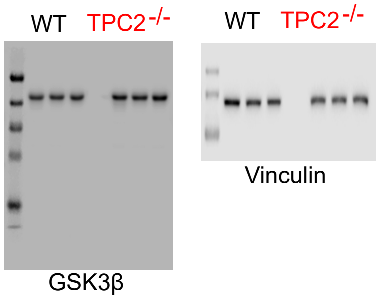

Fig.6k

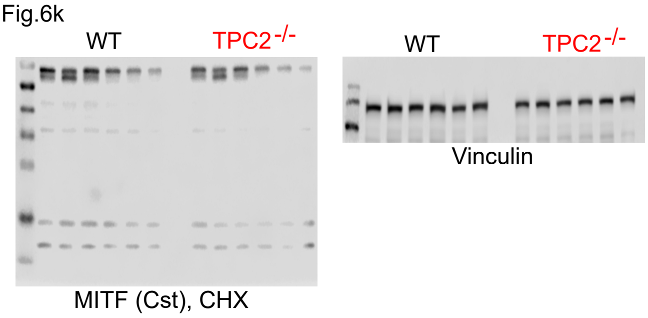

Fig.6n

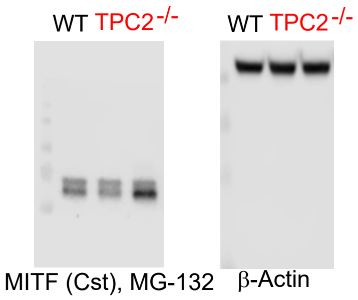

Suppl. Fig.S1a

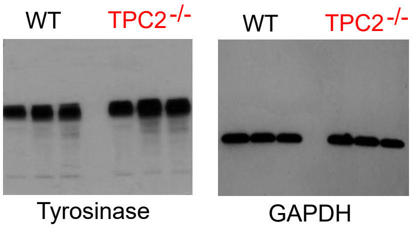

Suppl. Fig.S1e

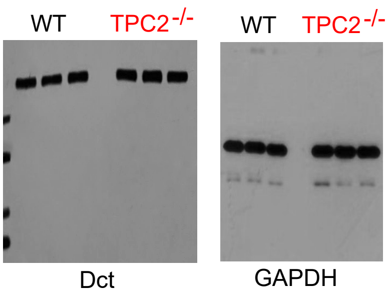

Suppl. Fig.S1c

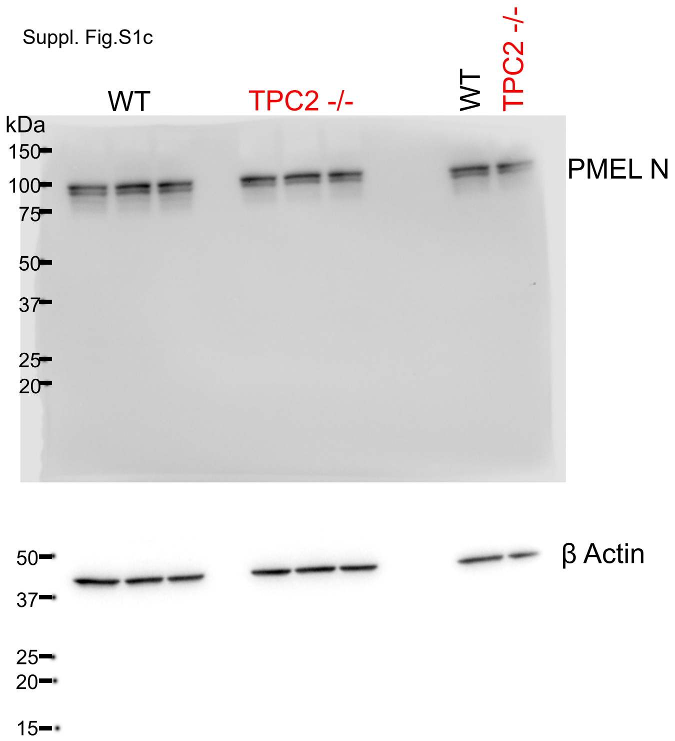

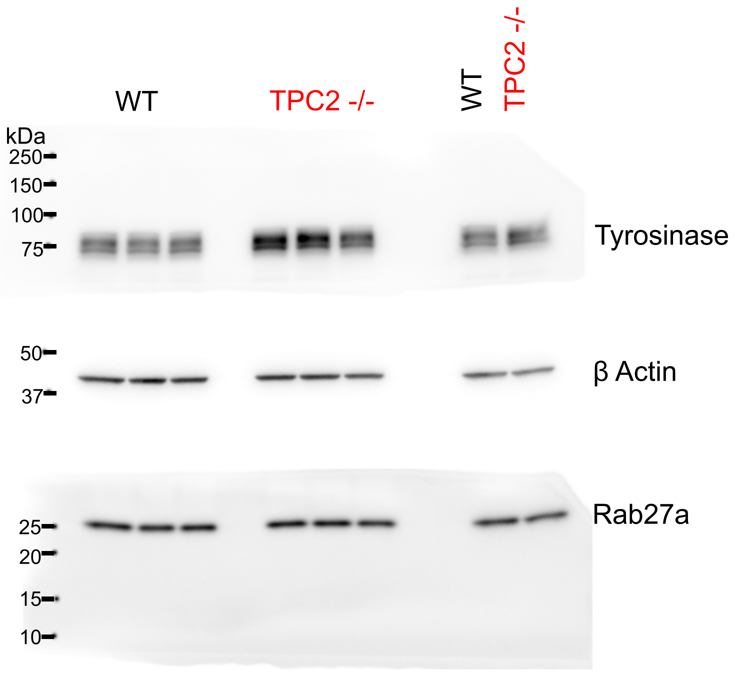

Supplement: Supplementary file 1 — Supplementary Information. [file 41598_2021_88196_MOESM1_ESM.pdf]
